# Monthly Meeting of the Medical Society of the County of Albany, Held January 9th, 1871

**Published:** 1871-02

**Authors:** James S. Bailey


ART. III.—*Monthly Meeting of the Medical Society of the County of Albany, held January 9th*, 1871.

REPORTED BY JAMES S. BAILEY, M. D.

Dr. Wm. H. Bailey, President, in the chair.

Dr. Amos Fowler reported a case of progressive paralysis.

June 16th, 1870. I was called to see Mr. M——, aged 56; his general appearance was healthy—was temperate in habit, except smoked incessantly.

In May, 1869, he spoke to me of a difficulty in retaining the saliva when reading or speaking. Two months later there was no improvement in this particular; and he complained of not feeling so well—he had difficulty in pronouncing some words, but thought the difficulty was in the throat. An examination revealed nothing.

In the Fall he was loosing his speech. He left town until June, when I was called. At this time his eyes seemed natural and bright, but there was but little expression to his face; the lower lip was inclined to drop, and the saliva was constantly dribbling. He could extend his tongue about half an inch, but could not articulate a word nor a letter plainly. Deglutition was so difficult that only some fluids could be swallowed; would often put his hands to his cheeks or mouth apparently to assist in swallowing; deglutition invariably produced coughing. There was pain most of the time in the back of the neck, and in the arms and legs. The the thumbs and arms were partially paralyzed, in connection with paresis of the tongue, lips and larynx. His gait, when walking, was dragging—was easily fatigued, and could walk but a few rods at a time. Could not raise a spoon containing fluid to his mouth, but could write a very legible hand. His memory was good, and his sight and hearing unimpaired; was free from headache; pulse 75; bowels a little torpid, and his stomach craved food.

The paralysis was progressive, and the muscular system gradually gave away; and, what is singular, notwithstanding the loss of he motor power in the muscles, and atrophy and degeneracy in all its parts, there was a morbid sensibility in all parts of the body. The moving of his hands or feet caused pain.

September 15. Was unable to leave his room, but could walk a little by being supported. The horizontal position caused a sense of suffocation, owing to the fact that the diaphragm performed the office of respiration mostly. The pectoral muscles being palsied as well as all the voluntary muscles. He was compelled to sleep in his chairs.

October 1st. He had lost the use of his hands and feet, and communicated his wants by a stick being placed between his fingers—he would point to letters on a newspaper to spell the words, and an attendant would write them as fast as produced.

October 20th. Had a severe chill, followed, in a day or two, by swelling in the left arm; the flexors of the arm and fingers became contracted; his tongue was immovable; his urine was scanty and loaded with phosphate; his urine was voluntarily passed, and he could also control the action of his bowels.

About the first of December the feet began to swell. December

30th, had an attack of short and difficult breathing almost resembling croup, which was relieved temporarily by morphine.

January 4th, he expired.

The question arises, where was the seat and cause of this gradual and insidious progression of symptoms, beginning with the lips, tongue, mouth and larynx and interfering with the functions of locomotion? Where is the anatomical site of the lesion that should cause this muscular atrophy and tissue change? Is it a degeneracy of the nerves, or its neuralemma, or is it in the spinal cord?

*Autopsy.*—The principle lesion was in almost the destruction of the roots of the hypoglossal nerves. The fifth pair of nerves were almost entirely obliterated, only a few fragments left.

The spinal accessory nerves could not be found. The anterior roots of the spinal cord were much atrophied. The brain seemed healthy, except was engorged with blood. The dura mater was thickened; the arachnoid was filled with serum.

Dr. R. H. Sabine presented an interesting specimen of hydatids of the liver and bladder.

The case was a patient of Dr. A. W. Shiland, who furnishes the following history.

Mr. ——, age 45. His health began to fail five years ago. Upwards of three years ago a tumor was discovered situated in the lower part of the abdomen, the pressure of it at times interfered with the passage of urine, and the patient complained of great pain in the right shoulder, which lasted several months.

During the last three months of his life it became necessary, occasionally, to use the catheter to draw off his urine.

About three months ago he was seized with a severe chill, which lasted for two hours, with violent pains in the left side, in the region of the liver and lower part of the right lung. About this time the physical signs gave evidence of solidification of two-thirds of the right lung. There was no cough, and there was not sufficient acceleration of the pulse to indicate inflammatory action. About this time the abdomen and lower extremities became dropsical, the difficulty in passing water increased, and his bowels were not moved except by cathartics or enemas.

*Post Mortem.*—The right lung was compressed by a sack containing five or six quarts of fluid. This sack rested upon the liver, and seemed to be developed from it. A large tumor was found, filling the cavity of the pelvis and pressing against the posterior portion of the bladder. This sack seemed to be formed from the outer coat of the bladder, and was adherent to the spine. It contained three or four quarts of hydatids of different sizes, from that of a pea to a large sized hen's egg. There were detached pieces of membrane floating inside of this sack, which resembled pieces of tripe, and were undoubtedly detached pieces of the internal membrane of this cyst. A microscopic examination of the contents of the hydatids exhibited animal parasites called echinoccoci.

There was an effusion of serum in the abdominal cavity.

Dr. Chas. A. Robertson then proceeded to read an elaborate and interesting paper entitled "Medical Ethics." At the close of which he remarked, he had only brought as much of his manuscript as he supposed they would have patience to hear.

Dr. Wm. H. Craig immediately moved that Dr. Robertson be requested to read the remainder of his paper at our next meeting, which was unanimously carried.

The Society took a recess for refreshments, after which it adjourned.

